# Unmet health and social service needs in LGBTQ+ older adults in California: a quantitative analysis of a large community-based sample

**DOI:** 10.3389/fpubh.2026.1931587

**Published:** 2026-10-08

**Authors:** Preetam Vupputuri, Angela K. Perone, Annesa Flentje, Nadra E. Lisha, Mira Terdiman, Ashwin A. Kotwal

**Affiliations:** 1 School of Public Health, University of California, Berkeley, Berkeley, CA, United States; 2 School of Medicine, University of California, San Francisco, San Francisco, CA, United States; 3 School of Social Welfare, University of California, Berkeley, Berkeley, CA, United States; 4 Stanford Prevention Research Center, Stanford University, Palo Alto, CA, United States

**Keywords:** equitable aging in health framework, health equity, health services accessibility, healthy aging, LGBTQIA+ aging, social determinants of health, unmet health and social service needs

## Abstract

**Background:**

LGBTQ+ older adults experience substantial unmet health and social service needs, yet little is known about whether chronological age is independently associated with unmet need or primarily reflects broader structural vulnerability. We examined the association between age and unmet health and social service needs among LGBTQ+ older adults using the Equitable Aging in Health Framework.

**Methods:**

We conducted a cross-sectional analysis of 3,713 LGBTQ+ adults aged 50 years and older participating in California’s first statewide survey of LGBTQ+ older adults. Any unmet health and social service need during the previous 12 months was the primary outcome. Sequential multivariable logistic regression models evaluated the association between chronological age and unmet need after adjustment for intersecting positionalities, social and political determinants of health, domains of power, and health status. Missing covariate data were addressed using multiple imputation by chained equations. Adjusted predicted probabilities and sensitivity analyses were also performed.

**Results:**

Overall, 36.6% of participants reported at least one unmet health or social service need. Greater chronological age was associated with lower odds of unmet need in unadjusted analyses [odds ratio (OR) per 10-year increase, 0.90; 95% confidence interval (CI), 0.83–0.97; *p* = 0.008], but this association was no longer statistically significant after full adjustment (OR, 1.08; 95% CI, 0.98–1.19; *p* = 0.100). Financial insecurity, transportation barriers, poorer physical health, depression, HIV-positive status, housing instability, caregiving support needs, lifetime trauma, and race and ethnicity remained associated with unmet need after multivariable adjustment, while chronological age did not.

**Conclusion:**

Chronological age was a relatively weak marker of unmet health and social service need among LGBTQ+ older adults, when considering broader social, structural, and health-related factors. Findings from one of the first quantitative applications of the Equitable Aging in Health Framework suggest that social and structural conditions may provide a more informative basis for understanding vulnerability than age alone, supporting more equitable approaches to aging policy, service delivery, and future research.

## 1 Introduction

LGBTQ+ older adults are particularly vulnerable to unmet health and social service needs (1, 2). Decades of stigma, exclusion, and trauma shape aging throughout the life course (3–5). These experiences contribute to disproportionately high rates of financial, social, and health-related vulnerability (1, 3, 6). Fear of discrimination and limited availability of LGBTQ+-inclusive resources remain common for adults in later life (1). These barriers contribute to elevated risk for unmet health and social needs and adverse effects that may follow (1).

Understanding vulnerability to unmet need is particularly important because many aging systems rely heavily on chronological age when determining eligibility for services and supports. Major public programs, including Medicare, Older Americans Act programs, Area Agencies on Aging services, and many state aging-service systems, use age-based thresholds to allocate resources and supports (7, 8). Yet the health and social service needs that shape people’s experience of aging span multiple interconnected domains, including healthcare, social support, transportation, housing, and other forms of assistance. However, little is known about whether chronological age functions as a meaningful marker of vulnerability to unmet need among LGBTQ+ older adults.

There are several reasons to question whether chronological age alone captures vulnerability within LGBTQ+ populations adequately. LGBTQ+ older adults have often navigated many years of minority stress, discrimination, and identity concealment (3–5). Exclusion from social institutions and unequal access to economic and social resources across the life course shape LGBTQ+ adults’ experience of aging (3–5).

Further, research on LGBTQ+ aging and healthcare access consistently identified stronger associations with social and structural factors than with chronological age alone. In the Caring and Aging with Pride Study (n = 2,560), health-related quality of life was more strongly associated with discrimination, chronic conditions, social support, and financial resources than with age (5, 9). Similar patterns were observed for health behaviors and healthcare barriers (10). Age was statistically associated with routine healthcare utilization, but the effect size was small (OR = 1.01) (11). In a separate survey of LGBTQ+ older adults, access to affirming healthcare providers was associated with greater uptake of preventive care and better chronic disease management (12). Together, these findings suggest that health and service-related outcomes among LGBTQ+ older adults may be more closely associated with social and structural conditions than with chronological age alone.

Despite extensive documentation of health disparities among LGBTQ+ older adults, relatively little research has explicitly examined whether chronological age is independently associated with unmet health and social service needs. Evidence from the broader non-LGBTQ+ aging literature is similarly mixed. A recent systematic review found that most studies reported no significant association between age and unmet healthcare need (44 of 61 studies), while 16 studies reported greater unmet need among younger older adults than among the oldest age groups (13). These findings suggest that chronological age may be an incomplete marker of vulnerability even within general aging populations. It also remains unclear whether apparent age-related differences in unmet need reflect chronological age itself or broader structural conditions associated with vulnerability.

A second gap relates to how service need has been conceptualized in LGBTQ+ aging research. Existing studies have focused primarily on healthcare utilization, healthcare barriers, preventive care, chronic disease management, and quality of life (9–12). Much less is known about broader patterns of unmet need spanning healthcare, behavioral health, transportation, housing, social support, caregiving, and aging-related services (9–12). Examining unmet service needs across these domains provides a more comprehensive view of unmet need beyond healthcare, without assuming that all services are equally severe or consequential. This broader perspective is particularly relevant to aging-service systems, which coordinate or connect older adults to multiple forms of health and social support rather than addressing a single service domain (7, 8). Whether chronological age meaningfully distinguishes vulnerability across this range of service needs remains unclear.

Understanding whether chronological age functions as a meaningful marker of vulnerability has important implications for aging policy and service delivery. Age-based eligibility systems rely on chronological age as an administratively useful tool to allocate services (7, 8). Although convenient, a growing body of literature suggests that chronological age may be an imperfect proxy for service need compared with measures such as frailty and functional status (14, 15). Yet whether age functions as a meaningful marker of unmet need within LGBTQ+ aging populations remains understudied, particularly when broader social and structural conditions are considered. Given the strong evidence placing LGBTQ+ older adults at elevated risk for unmet service needs and adverse aging outcomes, the lack of research examining how age operates within the broader web of structural determinants represents a critical barrier to designing equitable aging policies and services. Without a clearer understanding of whether age is associated with unmet need, aging service provision systems may overlook LGBTQ+ older adults most in need of services if they rely on age-based criteria alone.

Building on other foundational theories, including minority stress theory, the Equitable Aging in Health Framework (EAHF) offers a useful framework for addressing this gap (16). Minority stress theory serves as an important foundation for understanding how stigma, prejudice, discrimination, and related stress processes shape health among sexual and gender minority populations (17). The EAHF complements this perspective by foregrounding power and situating aging within the broader structural context that shapes access to resources, opportunities, services, and systems of support (16). As a cross-disciplinary and cross-methodological framework, the EAHF does not prescribe specific causal pathways or statistical relationships among domains. Instead, it offers a broader conceptual framework that allows for flexibility in examining complexity and intersections among determinants of health and domains of power. Its most important contribution is its explicit inclusion of domains of power, which are often more implicit in other frameworks. Factors such as race and ethnicity, financial insecurity, social support, and lifetime trauma may independently shape vulnerability by influencing both the need for services and the ability to access them. These are not divorced from systems of power, and EAHF provides conceptual grounding for exploring these connections. This framing is particularly important for LGBTQ+ communities, whose health and aging are shaped by cumulative social and structural exposures across the life course and longstanding sources of resilience and community strength (4, 5, 12, 18).

The EAHF served as the organizing framework for study design, variable selection, and model construction (16). Consistent with its flexible conceptual structure, we used the EAHF to identify and organize theoretically relevant covariates rather than to specify mediation or moderation pathways. Guided by the framework, we studied how the association between chronological age and unmet need changed as intersecting positionalities, social and political determinants of health, domains of power, and health status were sequentially incorporated into the models. This framework-driven approach allows age to be examined in the same manner that aging policy often uses it – as a primary lens for identifying vulnerability – while evaluating whether age remains associated with unmet need once accounting for broader dimensions of structural vulnerability.

To address these gaps, we analyzed data from California’s first statewide survey of LGBTQ+ older adults, a community-engaged needs assessment developed in partnership with LGBTQ+ community organizations, researchers, and the California Department of Aging. This analysis was designed in conversation with community partners and policymakers. A key priority was gaining a comprehensive understanding of unmet needs and access to aging-related services across age groups to inform future policy and program development. We sought to: (1) describe the prevalence and types of unmet health and social service needs among LGBTQ+ adults aged 50 years and older; (2) examine whether unmet need differed across age groups; and (3) evaluate the association between chronological age and unmet need using sequential models guided by the EAHF. Using a Population, Exposure, Comparator, and Outcome (PECO) framework, the population comprised LGBTQ+ California adults aged 50 years and older; the exposure was chronological age; the comparator was lower versus higher values of chronological age, modeled continuously; and the outcome was any unmet health or social service need. We hypothesized that chronological age would have a relatively weak independent association with unmet need after accounting for intersecting positionalities, social and political determinants of health, domains of power, and health status. In prespecified exploratory analyses informed by the EAHF, we also examined whether the association between chronological age and unmet need differed by HIV status, racial/ethnic minority status, financial insecurity, and housing instability.

## 2 Methods

### 2.1 Data and sample

This cross-sectional study used data from the first statewide survey of LGBTQ+ older adults in California: *From Challenges to Resilience*, conducted between January and March 2024, among LGBTQ+ adults aged 50 years and older living in California. The survey was developed through a community-engaged partnership involving Openhouse SF, the California Department of Aging, the University of California (UC), San Francisco, the Center for the Advanced Study of Aging Services at UC Berkeley, and other LGBTQ+ community organizations across the state. The study was guided by a statewide Advisory Committee including representatives from dozens of community organizations throughout California. The Advisory Committee identified research priorities and contributed to the conceptualization, design, recruitment, and dissemination of the study. Participants were recruited through outreach by LGBTQ+-serving and older adult-serving organizations using convenience sampling.

The original survey included 5,966 responses. Following eligibility and quality-control procedures conducted by the original survey team, including exclusion of 1,521 ineligible responses, 406 potential bot or fraudulent responses, and 2 trolling responses, the final dataset contained 4,037 observations. Potential bot or fraudulent responses were identified using Qualtrics RelevantID fraud scores (flagged when greater than or equal to 30) and reCAPTCHA measures (flagged when less than 0.5). Trolling responses were identified manually. For the present analysis, we excluded 127 surveys completed in less than 5 min and 16 respondents without chronological age data, leaving 3,894 respondents. An additional 181 respondents for whom the primary outcome could not be determined were excluded, yielding a primary analytic sample of 3,713 LGBTQ+ older adults. Missing covariate data within this cohort were addressed using multiple imputation as described below. Participant flow is shown in Supplementary Figure S1.

The original *From Challenges to Resilience* survey was approved by the University of California, San Francisco Institutional Review Board (IRB #23–38,987). All participants provided electronic informed consent before completing the survey.

### 2.2 Conceptual framework

Analyses were guided by the EAHF (16). The framework informed study design, covariate selection, and sequential model construction. Chronological age was selected as the primary exposure because eligibility for many aging-related programs, services, and public policies is determined using age-based thresholds (7, 8). Covariates were selected *a priori* based on the EAHF and organized into theoretically informed domains (Figure 1).

**Figure 1:**
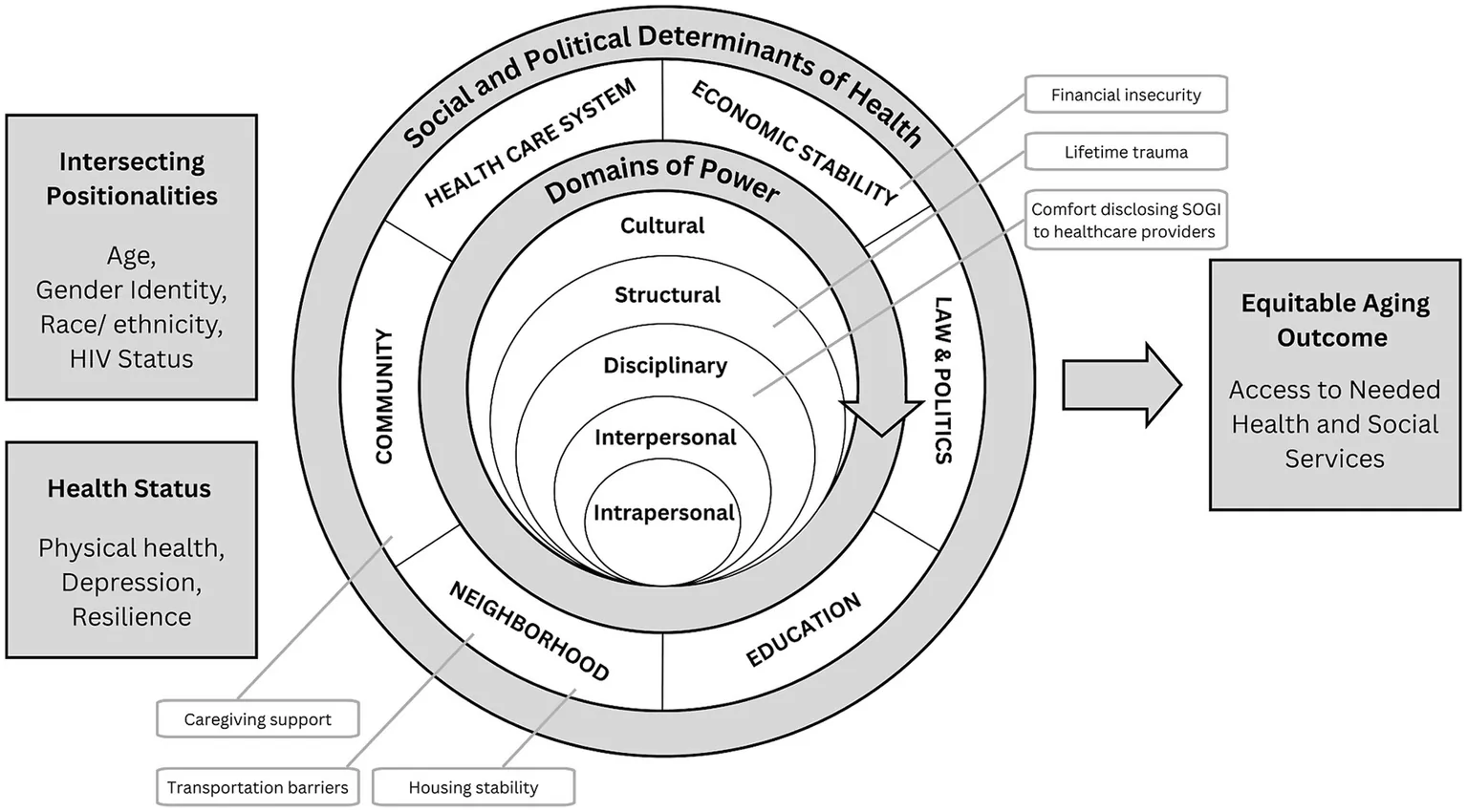
Equitable Aging in Health Framework (EAHF) conceptual model guiding study design and analysis. The EAHF situates unmet health and social service needs within intersecting structural, social, and individual determinants rather than chronological age alone. Chronological age was modeled as the primary exposure because age is commonly used to determine eligibility for aging services. Covariates were selected *a priori* and organized into four framework domains: intersecting positionalities, social and political determinants of health, domains of power, and health status. Variables included in each domain are shown in the surrounding boxes.

We conceptualized chronological age as an administratively important characteristic while recognizing that financial insecurity, housing instability, transportation barriers, HIV status, trauma, and health status may independently influence both access to services and unmet needs.

### 2.3 Measures

The primary exposure was chronological age, measured continuously in years and modeled per 10-year increase. For descriptive analyses only, participants were categorized into three age groups (50–59, 60–69, and 70 + years).

The primary outcome was any unmet health and social service need during the previous 12 months. Participants reported which services they needed and which services they used. An unmet need was defined as reporting a need for any service without reporting use of that service during the same period. Participants were classified as having an unmet need if they reported at least one unmet need across 21 health and social service categories. The service categories spanned healthcare, behavioral health, dental care, social support, legal, financial, transportation, housing, employment, caregiving, and aging-related services. For each check-all-that-apply question, unselected service options were treated as not endorsed only when evidence indicated that the question had been answered; complete question-level nonresponse was retained as missing. Selection of “None” and “Other” was considered evidence that the corresponding question had been answered, although these responses were not included among the 21 service categories. As such, respondents who answered the service-need and service-use questions but did not endorse an unmet service need were classified as having no unmet need, whereas respondents with insufficient information to determine unmet-need status due to question-level nonresponse were coded as missing.

The secondary outcome was unmet service count, defined as the total number of service categories for which participants reported needing but not receiving services.

Covariates represented four EAHF domains (Figure 1): intersecting positionalities (gender identity, race and ethnicity, HIV status); social and political determinants of health (financial insecurity, housing instability, transportation barriers, receipt of caregiving); domains of power (comfort disclosing sexual orientation and gender identity, SOGI, to providers and lifetime trauma); and health status (self-rated physical health, depression, and resilience). SOGI disclosure comfort and lifetime trauma were conceptualized as individual-level experiences through which broader relations of power may be encountered, rather than direct measures of structural power. Consistent with the EAHF’s conceptualization of intrapersonal and interpersonal domains of power as distinct but interrelated, comfort with disclosing SOGI may reflect experiences or expectations of stigma within healthcare, while lifetime trauma may capture adverse experiences occurring within broader social and structural contexts. Thus, these measures were not conceptualized as corresponding exclusively to a single level of power.

Depressive symptoms were assessed using the two-item Patient Health Questionnaire (PHQ-2; score range, 0–6), with scores greater than or equal to three classified as a positive depression screen (19). The PHQ-2 has demonstrated criterion and construct validity as a brief depression screening instrument, including in older adults (19, 20). Self-reported resilience was assessed using a single item adapted from the Brief Resilience Scale (BRS), “I tend to bounce back quickly after hard times,” which captures the perceived ability to recover from adversity (21). Because the full six-item BRS was not administered, this measure was interpreted as a single-item indicator of perceived resilience rather than the validated BRS. Detailed definitions and coding of all study variables are provided in Supplementary Table S1.

### 2.4 Statistical analysis

Analyses were conducted using R version 4.4.2. Statistical tests were two-sided with α = 0.05. No adjustments for multiple comparisons were made because the primary analysis evaluated a single prespecified exposure within a theory-informed model, and the interaction analyses were limited to a small set of prespecified, hypothesis-driven comparisons. Interaction findings were considered exploratory and interpreted cautiously.

Participant characteristics were summarized overall and by age group. The prevalence of unmet health and social service needs by service category and reported barriers to accessing needed services were also summarized descriptively overall and by age group. For descriptive analyses, age was categorized into three prespecified groups (50–59, 60–69, and 70 + years). Additional descriptive analyses compared service-specific unmet needs between White participants and participants of color.

Primary analyses were restricted to participants with observed chronological age and a calculable primary outcome (N = 3,713). Missing covariate data were addressed using multiple imputation by chained equations (MICE). Chronological age and the primary outcome were not imputed. Twenty imputed datasets were generated using 20 iterations, with conditional imputation models chosen according to variable type (22). Regression estimates and corresponding standard errors were pooled across imputed datasets using Rubin’s rules. Variable-specific missingness and imputation models are reported in Supplementary Table S3.

To evaluate the association between chronological age and unmet service needs, we fit a sequence of multivariable logistic regression models guided by the EAHF. Chronological age was modeled as a continuous variable, with odds ratios estimated per 10-year increase to improve interpretability while retaining the information within the continuous measure. Theory-based covariates were organized by EAHF domain and added sequentially: intersecting positionalities, followed by social and political determinants of health, domains of power, and health status. Sequential models were used to evaluate how the association between age and unmet need changed after accounting for distinct EAHF domains. The fully adjusted model, which included covariates from all four domains, was used for primary interpretation. Estimates were pooled across the 20 imputed datasets.

A final multivariable logistic regression model including all EAHF domains was used to estimate adjusted odds ratios (ORs) and 95% confidence intervals (CIs). Adjusted predicted probabilities of unmet need were estimated using average marginal predictions and pooled across the multiply imputed datasets. Omnibus *p* values for predictors in the fully adjusted model were obtained using pooled D1 tests.

The unmet-service count was overdispersed, with Pearson dispersion estimates from Poisson models ranging from 1.94 to 1.97 across imputed datasets. Therefore, negative binomial regression, which accommodates overdispersion in count outcomes, was used rather than Poisson regression (23). Model estimates were pooled across the multiply imputed datasets and reported as incidence rate ratios (IRRs) with 95% CIs.

Sensitivity analyses evaluated alternative specifications of chronological age and nonlinear age effects using restricted cubic splines. Prespecified interaction terms between age and HIV status, racial/ethnic minority status, financial insecurity, and housing instability were also evaluated separately as exploratory analyses informed by the EAHF. Additional analyses compared negative binomial and quasi-Poisson count models.

## 3 Results

### 3.1 Participant characteristics

The analytic sample included 3,713 LGBTQ+ adults aged 50 years and older (mean age 66.0 years, SD 8.3). Of participants, 24% were aged 50–59 years (*n* = 881), 42% were 60–69 years (*n* = 1,555), and 34% were 70 years and older (*n* = 1,277; Supplementary Table S2).

Overall, 1,360 participants (36.6%) reported at least one unmet health or social service need. The prevalence of any unmet need differed modestly across age groups, decreasing from 38.8% among adults aged 50–59 to 37.6% among those aged 60–69 and 33.9% among those aged 70 years or older (*p* = 0.038). The mean number of unmet needs also differed across age groups (1.0, 0.9, and 0.7, respectively; *p* < 0.001). Younger participants more frequently reported financial insecurity, housing instability, lifetime trauma, and screening above the cutoff for depression, whereas older participants more frequently reported receipt of caregiving and higher resilience. HIV status, race and ethnicity, gender identity, and comfort disclosing SOGI to healthcare providers also differed significantly across age groups (all *p* < 0.012). Transportation barrier frequency and self-rated physical health did not differ significantly across age groups (*p* = 0.079 and *p* = 0.50, respectively).

### 3.2 Prevalence and types of unmet service needs

Social support and dental services were the most reported unmet health and social service needs overall, reported by 10.4% (*N* = 386) and 10.1% (*N* = 376) of participants, respectively (Figure 2). Mental health or substance use services followed (6.3%, *N* = 233), then medical and health services (6.2%, *N* = 231), financial assistance or counseling (6.0%, *N* = 222), and legal assistance services (5.8%, *N* = 215). Social support and dental services remained among the most frequently reported unmet service categories across all age groups. Unmet needs for mental health or substance use services, financial assistance or counseling, and employment or job-seeking support were more frequently reported among younger participants, decreasing across successively older age groups. In contrast, unmet needs for aging services and information and referral services for seniors were more frequently reported among adults over 60 years than those aged 50–59 years. Supplementary Figure S2 shows the prevalence of specific unmet health and social service needs among white participants versus racial/ethnic minority participants.

**Figure 2:**
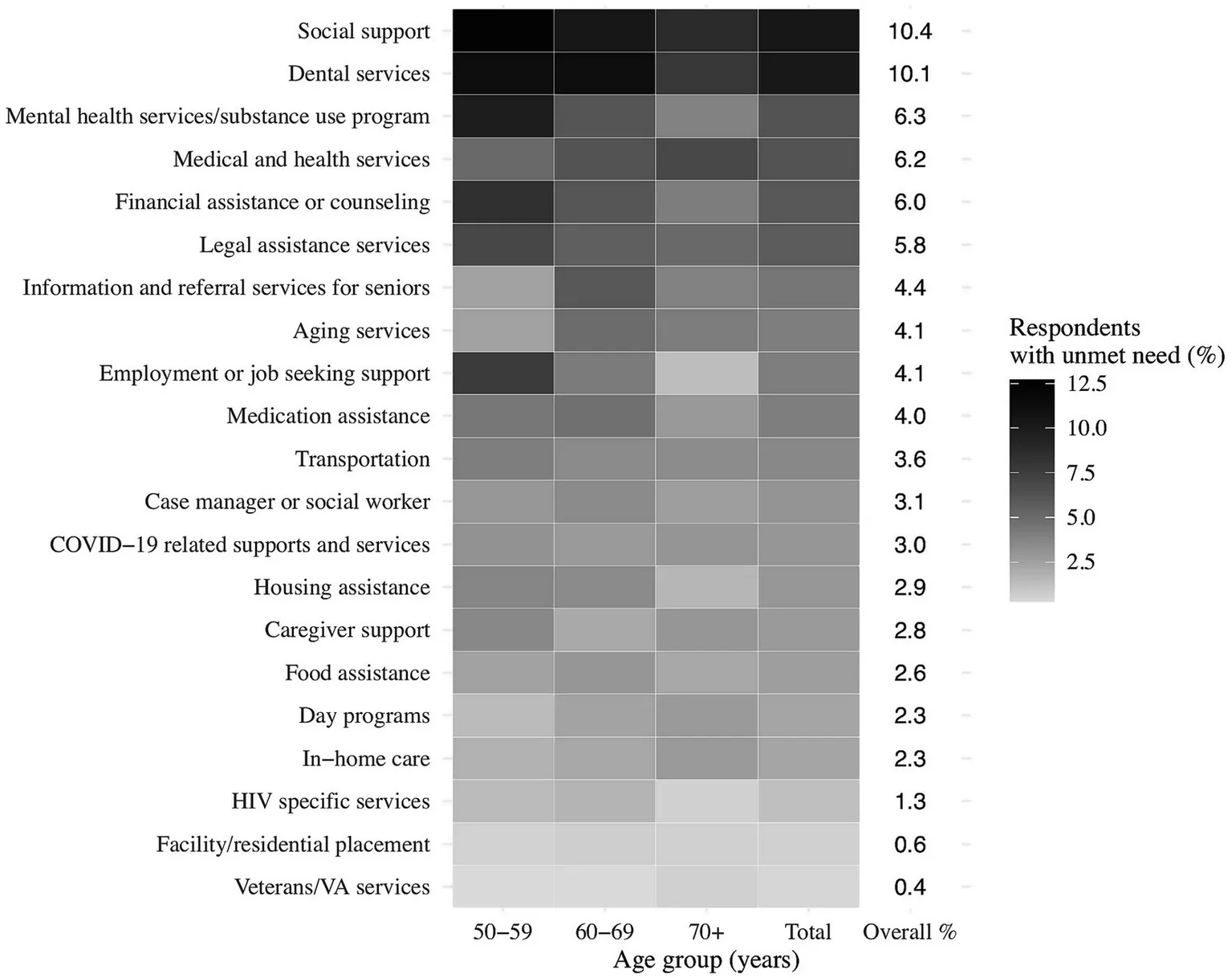
Prevalence of unmet health and social service needs by age group and overall. Service categories are ranked according to overall prevalence of unmet need across the analytic sample. Cell shading represents the percentage of respondents within each age group reporting an unmet need for that service category, with darker shading indicating greater prevalence. Values in the rightmost column represent overall prevalence across all participants.

### 3.3 Barriers to accessing needed services

Participants reported a range of barriers to obtaining needed services (Figure 3). Of all the reported barriers associated with unmet service need, the most frequent were difficulty accessing services (27.1%), concerns about eligibility (19.8%), and cost (15.8%). Barriers related to inclusivity and prior experiences were also common, including services that were not LGBTQIA+-friendly (9.5%), prior mistreatment (7.3%), and ageism or feeling unwelcome (6.5%). The ranking of barriers was broadly similar across age groups.

**Figure 3:**
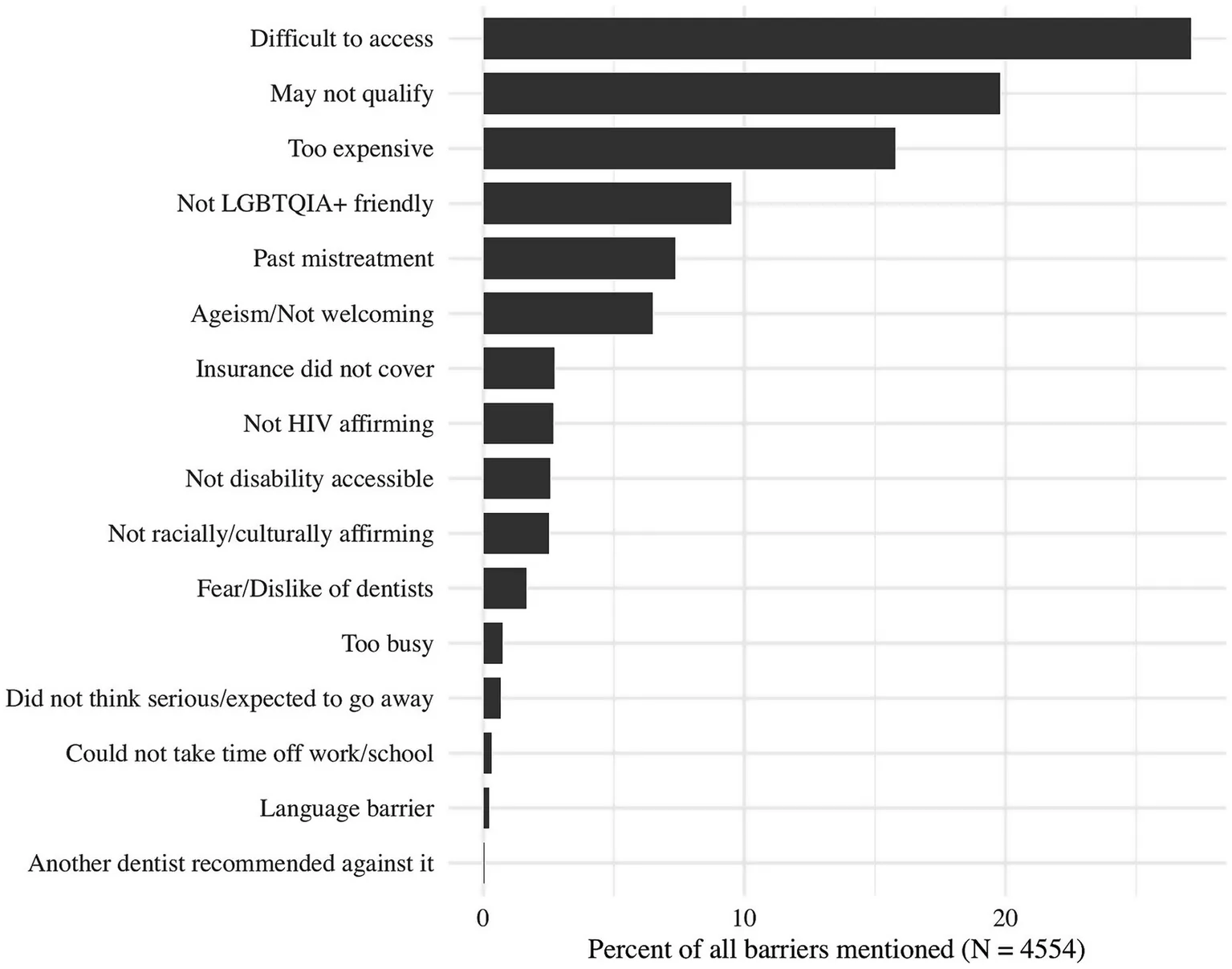
Reported barriers to accessing needed health and social services. Bars represent the percentage of all reported barriers among respondents who experienced at least one unmet health or social service need. Participants could select multiple barriers; therefore, the number of barriers mentioned is 4,554.

Among participants with unmet needs, 18.9% of participants of color versus 10.1% of White participants reported that services were not racially or culturally affirming. Similarly, services that were not HIV affirming were reported by 19.9% of HIV-positive participants compared with 5.7% of HIV-negative participants. Finally, 27.8% of transgender and gender-diverse participants (TGD) compared to 15.7% of non-TGD participants reported that services were not LGBTQIA+-affirming.

### 3.4 Association of age with unmet need across sequential EAHF models

Figure 4 presents the association between chronological age and unmet need across sequential EAHF models. In the unadjusted model, each 10-year increase in age was associated with lower odds of unmet need (OR per 10-year increase 0.90, 95% CI 0.83–0.97; *p* = 0.008). The estimated association was attenuated after incorporating intersecting positionalities (gender identity, race and ethnicity minority status, and HIV status) and changed direction as additional EAHF domains were included. In the fully adjusted model, chronological age was no longer significantly associated with unmet need (OR per 10-year increase 1.08, 95% CI 0.98–1.19; *p* = 0.100). Missing covariate data and multiple imputation methods are provided in Supplementary Table S3, and sequential model estimates are provided in Supplementary Table S4.

**Figure 4:**
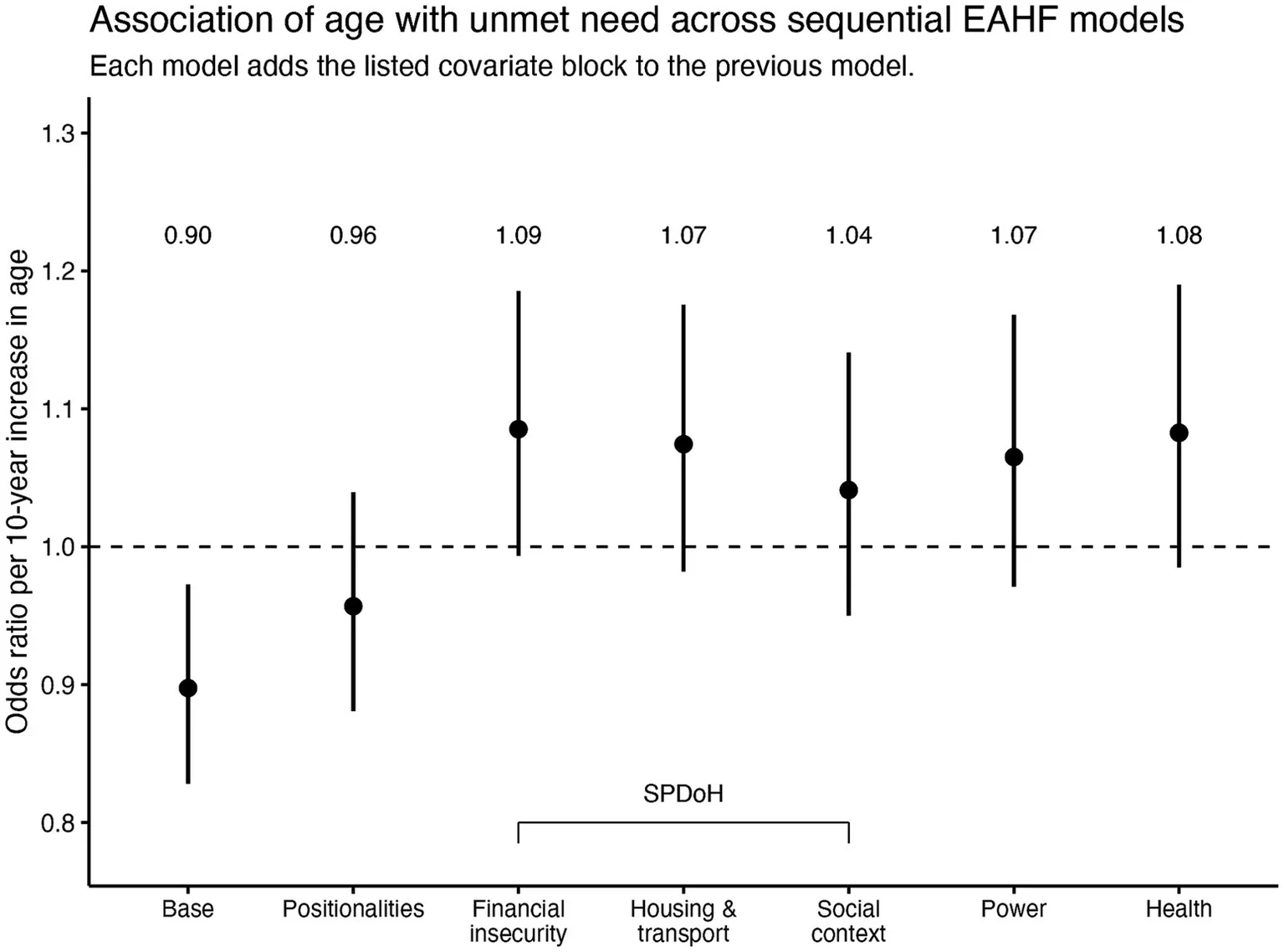
Association of age with unmet need across sequential Equitable Aging in Health Framework (EAHF) models. Points represent odds ratios (ORs) for any unmet need associated with each 10-year increase in age. Error bars represent 95% confidence intervals. Covariate domains were added sequentially from left to right according to the EAHF to evaluate how adjustment for intersecting positionalities, social and political determinants of health, domains of power, and health status changed the estimated association between age and unmet need. Estimates were pooled across 20 multiply imputed datasets. The dashed horizontal line indicates an OR of 1.0.

In prespecified exploratory interaction analyses, there was evidence of an interaction between chronological age and race and ethnicity minority status (interaction OR 1.30, 95% CI 1.04–1.63; *p* = 0.021). The interaction between age and HIV status was not statistically significant (pooled omnibus *p* = 0.116). There was also no evidence of interaction between age and financial insecurity (interaction OR 0.86, 95% CI 0.70–1.06; *p* = 0.165) or housing instability (interaction OR 0.90, 95% CI 0.68–1.18; *p* = 0.444).

### 3.5 Adjusted probabilities from the final EAHF model

Figure 5 presents adjusted predicted probabilities for characteristics associated with any unmet health and social service need in the fully adjusted EAHF model. Higher adjusted probabilities of unmet need were observed among participants reporting financial insecurity, frequent transportation barriers, poorer physical health, a positive depression screen, housing instability, HIV-positive status, caregiving support receipt, lifetime trauma, and among racial/ethnic minority participants. Adjusted probabilities also differed across resilience levels. Gender identity and comfort disclosing SOGI to providers were not associated with significant differences in adjusted probabilities. Complete adjusted predicted probabilities for all model covariates are shown in Supplementary Table S5.

**Figure 5:**
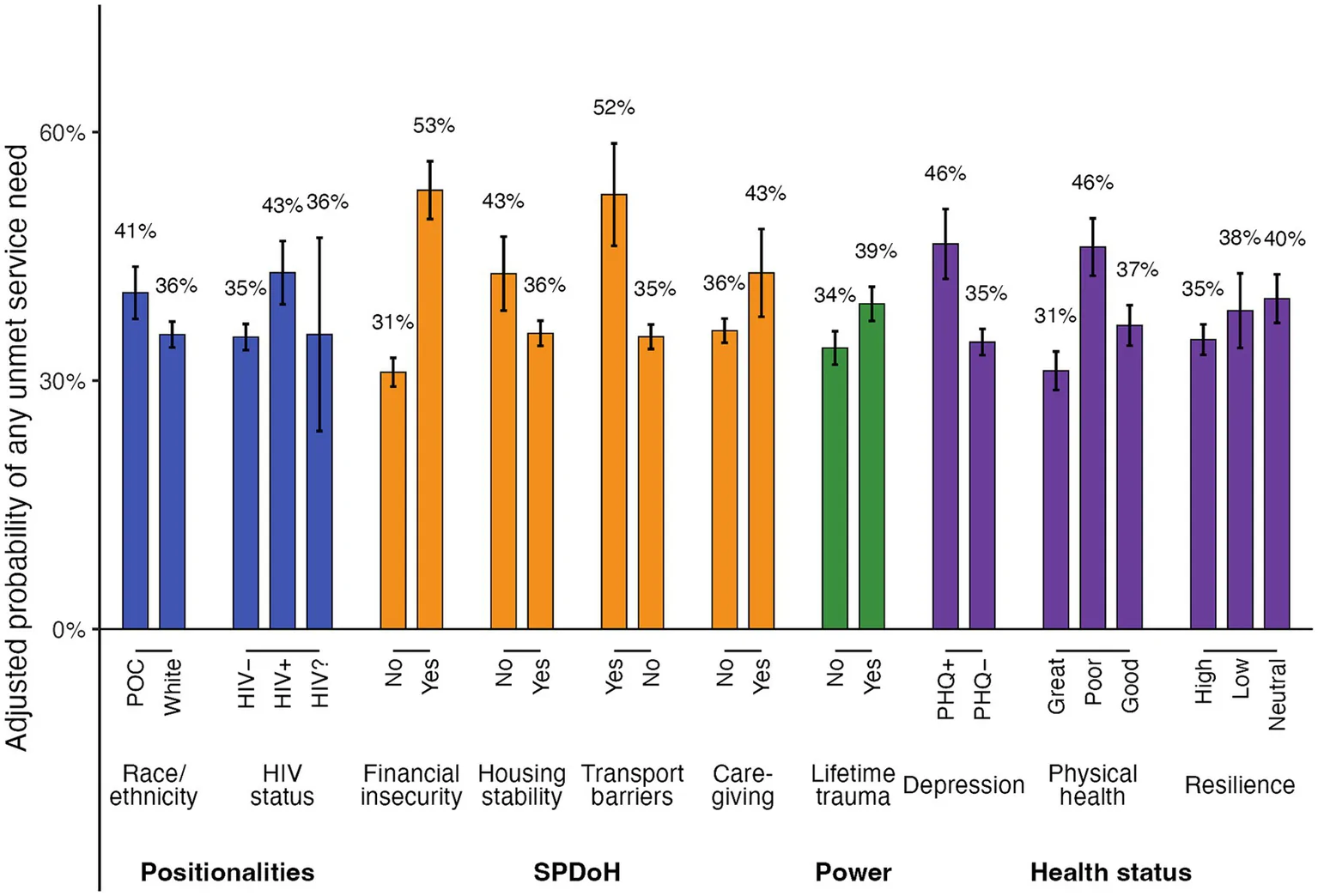
Adjusted predicted probabilities of unmet health and social service need for covariates significantly associated with unmet need in the fully adjusted Equitable Aging in Health Framework (EAHF) model. Predicted probabilities were estimated using average marginal predictions pooled across 20 multiply imputed datasets after adjustment for all covariates included in the final multivariable logistic regression model. Error bars represent 95% confidence intervals. The complete adjusted predicted probabilities are provided in Supplementary Table S5. Reference categories were White race and ethnicity, HIV-negative status, no financial insecurity, stable housing, no transportation barriers, no caregiving support, no lifetime trauma, lower depressive symptoms, excellent or very good self-rated physical health, and high self-reported resilience.

### 3.6 Secondary and sensitivity analyses

Results were generally consistent when unmet service burden was modeled as a count outcome using negative binomial regression (Supplementary Table S6). Chronological age was modestly associated with a greater number of unmet service needs (IRR per 10-year increase 1.07, 95% CI 1.00–1.15; *p* = 0.044). Financial insecurity, transportation barriers, HIV-positive status, housing instability, lifetime trauma, a positive depression screen, poorer physical health, and lower resilience remained associated with a greater number of unmet needs, while caregiving support network and comfort disclosing SOGI were not.

Results were also robust to alternate specifications of chronological age and missing-data handling. In a complete-case analysis (*N* = 3,242 from *N* = 3,713), the association between age and any unmet service need was similar to that observed in the primary multiply imputed analysis (OR per 10-year increase, 1.08, 95% CI 0.98–1.20; *p* = 0.131 vs. OR 1.08, 95% CI 0.98–1.19; *p* = 0.100, respectively). Alternative categorical specifications of age (50–59, 60–69, and 70 + years; 50–59 vs. 60 + years; 50–64 vs. 65 + years) did not materially change the overall interpretation. Restricted cubic spline models provided no evidence that a nonlinear specification improved model fit compared with a linear age specification (pooled D1 *p* = 0.207; Supplementary Figure S3). In a sensitivity analysis of the count outcome, quasi-Poisson regression yielded a directionally similar estimate for age (IRR 1.04, 95% CI 0.98–1.10; *p* = 0.244), supporting the consistency of the association’s direction and magnitude across count-model specifications.

## 4 Discussion

In this first statewide study of unmet health and social service needs among LGBTQ+ older adults in California, we found that chronological age was a relatively weak and inconsistent marker of unmet need. 36.6% of participants reported at least one unmet health or social service need, and although prevalence differed significantly across age groups, the magnitude of the difference was relatively small (38.8%, 37.6%, and 33.9% for 50–59, 60–69, and 70 + age groups respectively). Throughout sequential EAHF models, chronological age demonstrated little independent association with unmet need. Although chronological age was not significantly associated with the primary binary outcome after multivariate adjustment, it was modestly associated with the number of unmet service needs in the secondary count analysis. This suggests that the relationship between chronological age and unmet need may depend on how unmet service need is operationalized.

Although age remains an administratively important measure for determining eligibility for many aging services (7, 8), the broader gerontology literature has increasingly recognized chronological age is often a weaker predictor of health outcomes and service needs than measures of frailty, multimorbidity, and functional status (14, 15). Similarly, previous LGBTQ+ aging research has shown that healthcare utilization, barriers, and quality of life are more consistently explained by discrimination, social support, identity affirmation, and socioeconomic conditions than by chronological age itself (5, 9–11). Our findings extend this literature: after adjusting for domains of the EAHF, the association between chronological age and unmet health and social service needs was relatively modest across measures spanning healthcare, behavioral health, transportation, housing, social support, caregiving, and aging-related services among LGBTQ+ older adults. This composite approach was intended to capture the breadth of unmet service need experienced across interconnected domains rather than treating any single service category as the sole indicator of unmet need.

A key contribution of this study is its quantitative application of the EAHF. Beyond providing a theory-based way to operationalize and evaluate age-based assumptions in aging policy, the EAHF helps contextualize our findings by situating chronological age within the intersecting social, economic, political, and health-related systems in which vulnerability in later life is experienced (16). To our knowledge, this is the first quantitative application of the EAHF to examine unmet health and social service need among LGBTQ+ older adults using a sequential, theory-informed modeling approach. Rather than only asking whether chronological age matters, our findings suggest that a more informative question is which social and structural conditions shape vulnerability across LGBTQ+ older adult populations. While our study’s cross-sectional design could not establish such causality, future research could examine these relationships more explicitly.

Approximately two in five participants reported at least one unmet need, highlighting the substantial burden of unmet health and social service needs within this statewide LGBTQ+ aging population. Social support and dental care were the most frequently reported unmet service categories, while barriers related to access, affordability, eligibility, and LGBTQ+ affirming services were most common across age groups. These descriptive findings complement previous research documenting persistent structural barriers to healthy aging among LGBTQ+ older adults and underscore that unmet need extends beyond healthcare utilization to include broader social supports (1, 24, 25). Although several structural and health-related characteristics – including financial insecurity, transportation barriers, housing instability, and poorer health – were associated with unmet health and social service need in the fully adjusted model, models designed specifically to test these associations need to be further explored.

The observed interaction between age and the race and ethnicity variable also warrants additional investigation. Although chronological age demonstrated little association with unmet need overall, the association between age and unmet need was more positive among racial and ethnic minority participants than among White participants. This pattern could be consistent with theories of cumulative disadvantage and weathering, which suggest that structural inequities accumulate across the life course for racial and ethnic minorities (26, 27). California’s Master Plan on Aging explicitly foregrounds equity throughout its lengthy document; however, specific attention to intersections among sexual orientation, race and ethnicity, and age remains less clear (28); these findings underscore the importance of targeted programs and policies that explicitly address the needs of LGBTQ+ older adults with intersecting positionalities, including Black same-gender loving older adults and older LGBTQ+ immigrants.

These findings have several implications for aging policy and service delivery. Aging service systems should consider not relying solely on chronological age to identify individuals most likely to experience unmet health and social service needs. Instead, more comprehensive approaches that consider broader structural barriers, social determinants of health, and health-related dimensions of vulnerability may better identify LGBTQ+ older adults at greatest risk. This recommendation aligns with recent policy efforts in California to increasingly incorporate functional need, disability, or level-of-care criteria into aging and long-term care eligibility instead of relying on chronological age alone (28). Moreover, the reported unmet needs for dental and social care suggest that more attention is needed to support access to dental care and social services for LGBTQ+ older adults. Policies that protect and expand comprehensive Medi-Cal (California’s Medicaid) dental coverage and social services are needed as well as policy efforts that make oral health and social prescribing explicit components of California’s aging services infrastructure, such as through its Master Plan on Aging or by incentivizing Area Agencies on Aging to incorporate oral health into their services (28). These findings also support efforts to improve LGBTQ+-affirming care environments through provider education, culturally responsive service delivery, stronger partnerships between LGBTQ+ organizations and aging-service networks, and interventions to reduce social isolation and strengthen community connectedness (1, 12, 25, 29, 30).

This study has several limitations. The cross-sectional design precludes causal inference, and convenience sampling through LGBTQ+ and older adult organizations may have introduced selection bias and limited generalizability. Individuals experiencing unmet needs may have been more motivated to participate, inflating the observed estimates of unmet need. Conversely, perhaps organization-based recruitment preferentially reached individuals already connected to community service resources, resulting in lower estimates. The survey may also underrepresent LGBTQ+ older adults disconnected from community organizations, limited in their digital access, experiencing unstable social or economic circumstances, or facing other barriers to survey participation. Namely, some of the same social and structural conditions associated with service access may have also influenced participation, and the observed prevalence should not be interpreted as representative of all LGBTQ+ older adults in California.

Self-reported measures about the past 12 months may be subject to recall and reporting bias and could not be independently verified. The primary outcome was designed to capture any unmet need across 21 health and social service categories but did not capture the severity or acuity of individual needs. Resilience was assessed using a single item rather than the full validated BRS, potentially limiting measurement of the broader construct. Additionally, relatively small numbers of participants in some racial, ethnic, and gender-diverse subgroups limited more detailed subgroup analyses to collapsed POC and transgender/gender-expansive categories. Future research should examine unmet needs within specific service categories and priority populations, including people living with HIV and racial and ethnic minority communities, with sufficient sample sizes.

Despite these limitations, this study has several strengths. It is the first statewide quantitative analysis of unmet needs for LGBTQ+ older adults in California. It was developed through an extensive community-engaged partnership involving LGBTQ+ organizations, researchers, and state partners. The breadth of the survey allowed unmet need to be examined across health and social service domains that are often studied separately. The study also provides the first quantitative application of the EAHF to our knowledge. By examining chronological age within a theory-driven framework rather than as an isolated characteristic, this study contributes evidence that may inform future research and more equitable approaches to aging policy.

In conclusion, chronological age was a relatively weak and inconsistent marker of unmet health and social service need among LGBTQ+ older adults in California. Although unmet need was common, associations with chronological age varied across analytic approaches: age was not significantly associated with the primary any unmet need variable but was modestly associated with the number of unmet needs in the secondary count analysis. These findings suggest that chronological age alone may be insufficient for identifying LGBTQ+ older adults most likely to experience unmet need and support continued efforts to develop aging policies and services that recognize the broader social and structural contexts in which aging occurs.

## Data Availability

The data analyzed in this study is subject to the following licenses/restrictions: Deidentified survey data from the From Challenges to Resilience study are publicly available through the California Department of Aging. The continuous age variable used in this analysis was available only through the restricted research dataset under the approved IRB protocol. Information about the survey and publicly available resources can be found at https://aging.ca.gov/survey_of_LGBTQIA/. Researchers interested in accessing the restricted research dataset should contact the study investigators regarding data access requirements. Requests to access these datasets should be directed to https://aging.ca.gov/survey_of_LGBTQIA/ or Nadra Lisha, Nadra.Lisha@ucsf.edu.
