# Supplementary material for: Unmet health and social service needs in LGBTQ+ older adults in California: a quantitative analysis of a large community-based sample
Source: Front Public Health. 2026 Oct 8;14:1931587. doi: 10.3389/fpubh.2026.1931587 (PMC13686891; doi:10.3389/fpubh.2026.1931587)
Supplement: Supplementary file 1 [file Supplementary_file_1.DOCX]

Supplementary Material

**Supplementary Table S1. Definitions and coding of study variables.** Definitions, survey wording, and analytic coding of study variables used in analyses.

| Domain | Variable | Survey wording / derivation | Analytic coding |
| --- | --- | --- | --- |
| Age/ exposure | Continuous age (years) | Age obtained from a linked age file | Continuous age in years; retained for regression models |
|  | Age group | Age category (mutually exclusive) | Factor with levels 50-59, 60-69, 70+ |
| Intersecting Positionalities | Gender identity | *What is your current gender identity? (Check all that apply.)* | Recoded to: Cisgender man, Cisgender woman, Transgender man, Transgender woman, Gender expansive female Sex Assigned at Birth, Gender expansive Male Sex Assigned at Birth |
|  | Race/ethnicity subgroup indicator | Derived from race/ethnicity items | Binary grouping used for subgroup figure: People of Color (POC) vs White. Racial and ethnic minority status and POC terminology are used interchangeably. |
|  | Race/ethnicity | Derived from check-all-that-apply race/ethnicity items | Mutually exclusive analytic categories: White, Black/African American, Latine/Hispanic, Asian, American Indian/Alaska Native, Middle Eastern/North African, Native Hawaiian/Pacific Islander, Multiracial, Other/Self-described |
|  | HIV status | *What is your HIV status?* | Recoded to Negative, Positive, or Unknown (includes “Don’t know” and “Prefer not to disclose”) |
| Social and political determinants | Financial insecurity (past 12 months) | *Financially insecure* | Collapsed to Yes/Maybe, No, Missing |
|  | Housing stability | *What is your housing situation today?* | Collapsed to Stably Housed, Insecure/Unhoused, Missing |
|  | Transportation barrier frequency | *How often were you unable to get where you needed to go because you did not have transportation?* | Collapsed to Rare/Infrequent, Frequent, Missing |
|  | Caregiving/support network | Derived from number of people participants reported receiving care from | Collapsed to No (no reported caregiver/support received), Yes (greater than or equal to 1 caregiver/support reported), Missing |
| Domains of power | Comfort disclosing SOGI to provider | Derived from comfort with healthcare provider knowing sexual orientation and gender identity/sex assigned at birth | Collapsed to Comfortable, Neutral, Uncomfortable, Missing |
|  | Lifetime trauma experience | *Sometimes things happen to people that are unusually or especially frightening, horrible, or traumatic. For example: a serious accident or fire, a physical or sexual assault or abuse, an earthquake or flood, a war, seeing someone be killed or seriously injured, having a loved one die through homicide or suicide.*  *Have you experienced this kind of event?* | Collapsed to Yes, No, Missing |
| Baseline health status | Self-rated physical health | *In general, how would you rate your physical health?* | Collapsed to Excellent/Very Good, Good, Fair/Poor, Missing |
|  | Depression screen (above cutoff) | Percentage above and below the cutoff score for Major Depressive Disorder using the PHQ2 scale | Collapsed to Above Cutoff (Yes; PHQ score of 3+), Below Cutoff (No; PHQ score under 3), Missing |
|  | Resilience | *I tend to bounce back quickly after hard times.* | Collapsed to High (Agree), Neutral, Low (Disagree), Missing |
| Primary and Secondary Service Outcomes | Any unmet service need | Derived from service need and service use items | Binary outcome indicating ≥1 unmet need across 21 prespecified service categories during the past 12 months. An unmet need was defined as a service reported as needed but not used during the past 12 months among respondents who answered both service questions. Respondents whose unmet-need status could not be calculated due to question-level nonresponse were coded as missing. |
|  | Count of unmet needs | Derived from service need and service use items | Count of services needed but not used, using the same service-level definition as the primary outcome. |
| Services | Service need / service use items | *In the past 12 months, which of the following services did you need / actually use? (Check all that apply.)* | For each of 21 prespecified service categories was coded as endorsed (1) or not endorsed (0) within the corresponding check-all-that-apply question. Unselected options were coded as mot endorsed when the question was answered. “None” and “Other” were not included among the 21 service categories. |
| Barriers | Reasons for unmet care | *In the past 12 months, what were the reasons for not using the services that you needed? (Check all that apply.)* | Each barrier coded as endorsed (1) or not endorsed (0); dental services used a service-specific barrier item set |

Abbreviations: EAHF, Equitable Aging in Health Framework; PHQ-2, Patient Health Questionnaire-2; POC, People of Color; SAAB, sex assigned at birth; SOGI, sexual orientation and gender identity.

**Supplementary Figure S1. Participant flow from original survey responses to the descriptive and primary analytic cohorts.** The original survey contained 5,966 responses. Following survey team quality control procedures, 4,037 observations were included in the publicly available dataset. We subsequently excluded 127 surveys completed in less than 5 minutes and 16 respondents without age data, yielding 3,894 participants. An additional 181 respondents without an evaluable primary outcome were excluded from the primary analytic cohort, yielding a final 3,713 participants. Missing covariate data within the primary analytic cohort were addressed using multiple imputation.

# Supplementary Table S2. Participant characteristics of LGBTQ+ older adults overall and by age groups (N = 3,713). Values are n (%) or mean (SD). Percentages for categorical variables were calculated among participants with non-missing data for the corresponding characteristic; missing values are reported separately as n. The analytic sample was restricted to participants with calculable unmet-need status. P values compare age groups using Pearson’s χ² tests for categorical variables and Welch’s one-way ANOVA for continuous variables, as appropriate.

| **Characteristic** | **Overall N = 3,713*^1^*** | **50-59 N = 881*^1^*** | **60-69 N = 1,555*^1^*** | **70+ N = 1,277*^1^*** | **p-value*^2^*** |
| --- | --- | --- | --- | --- | --- |
| **Continuous age (years)** | 66.0 (8.3) | 55.2 (2.8) | 64.5 (2.9) | 75.2 (4.4) | <0.001 |
| **Any unmet service need** | 1,360 (37%) | 342 (39%) | 585 (38%) | 433 (34%) | 0.038 |
| **Count of unmet needs** | 0.9 (1.7) | 1.0 (1.8) | 0.9 (1.8) | 0.7 (1.4) | <0.001 |
| **Gender identity** |  |  |  |  | <0.001 |
| Cisgender man | 2,123 (57%) | 462 (53%) | 925 (60%) | 736 (58%) |  |
| Cisgender woman | 1,181 (32%) | 263 (30%) | 488 (31%) | 430 (34%) |  |
| Transgender man | 40 (1.1%) | 17 (1.9%) | 15 (1.0%) | 8 (0.6%) |  |
| Transgender woman | 60 (1.6%) | 18 (2.0%) | 20 (1.3%) | 22 (1.7%) |  |
| Gender expansive F SAAB*^3^* | 187 (5.0%) | 89 (10%) | 60 (3.9%) | 38 (3.0%) |  |
| Gender expansive M SAAB*^3^* | 115 (3.1%) | 31 (3.5%) | 43 (2.8%) | 41 (3.2%) |  |
| Missing | 7 | 1 | 4 | 2 |  |
| **Race/ethnicity** |  |  |  |  | <0.001 |
| White | 2,918 (79%) | 606 (69%) | 1,233 (79%) | 1,079 (84%) |  |
| Black/African American | 105 (2.8%) | 39 (4.4%) | 38 (2.4%) | 28 (2.2%) |  |
| Latine/Hispanic | 175 (4.7%) | 65 (7.4%) | 81 (5.2%) | 29 (2.3%) |  |
| Asian | 104 (2.8%) | 38 (4.3%) | 39 (2.5%) | 27 (2.1%) |  |
| American Indian/Alaska Native | 17 (0.5%) | 5 (0.6%) | 10 (0.6%) | 2 (0.2%) |  |
| Middle Eastern/North African | 17 (0.5%) | 12 (1.4%) | 4 (0.3%) | 1 (<0.1%) |  |
| Native Hawaiian/Pacific Islander | 6 (0.2%) | 3 (0.3%) | 2 (0.1%) | 1 (<0.1%) |  |
| Multiracial | 284 (7.7%) | 92 (10%) | 120 (7.7%) | 72 (5.6%) |  |
| Other/Self-described | 84 (2.3%) | 20 (2.3%) | 26 (1.7%) | 38 (3.0%) |  |
| Missing | 3 | 1 | 2 | 0 |  |
| **HIV status** |  |  |  |  | 0.004 |
| Negative | 2,894 (81%) | 685 (81%) | 1,174 (79%) | 1,035 (84%) |  |
| Positive | 620 (17%) | 145 (17%) | 296 (20%) | 179 (15%) |  |
| Unknown | 54 (1.5%) | 17 (2.0%) | 21 (1.4%) | 16 (1.3%) |  |
| Missing | 145 | 34 | 64 | 47 |  |
| **Financial insecurity (past 12 months)** |  |  |  |  | <0.001 |
| No | 2,753 (75%) | 570 (65%) | 1,137 (73%) | 1,046 (82%) |  |
| Yes/Maybe | 942 (25%) | 304 (35%) | 411 (27%) | 227 (18%) |  |
| Missing | 18 | 7 | 7 | 4 |  |
| **Housing stability** |  |  |  |  | <0.001 |
| Stably Housed | 3,222 (87%) | 734 (84%) | 1,344 (87%) | 1,144 (90%) |  |
| Insecure/Unhoused | 477 (13%) | 145 (16%) | 205 (13%) | 127 (10.0%) |  |
| Missing | 14 | 2 | 6 | 6 |  |
| **Transportation barrier frequency** |  |  |  |  | 0.079 |
| Rare/Infrequent | 3,303 (91%) | 762 (90%) | 1,388 (92%) | 1,153 (92%) |  |
| Frequent | 311 (8.6%) | 89 (10%) | 125 (8.3%) | 97 (7.8%) |  |
| Missing | 99 | 30 | 42 | 27 |  |
| **Caregiving/support network** |  |  |  |  | <0.001 |
| Yes | 301 (8.5%) | 53 (6.4%) | 91 (6.1%) | 157 (13%) |  |
| No | 3,256 (92%) | 776 (94%) | 1,396 (94%) | 1,084 (87%) |  |
| Missing | 156 | 52 | 68 | 36 |  |
| **Comfort disclosing SOGI to provider** |  |  |  |  | <0.001 |
| Comfortable | 3,118 (90%) | 683 (86%) | 1,315 (91%) | 1,120 (92%) |  |
| Neutral | 147 (4.3%) | 33 (4.2%) | 57 (3.9%) | 57 (4.7%) |  |
| Uncomfortable | 193 (5.6%) | 74 (9.4%) | 72 (5.0%) | 47 (3.8%) |  |
| Missing | 255 | 91 | 111 | 53 |  |
| **Lifetime trauma experience** |  |  |  |  | <0.001 |
| No | 1,775 (49%) | 472 (56%) | 778 (52%) | 525 (42%) |  |
| Yes | 1,821 (51%) | 371 (44%) | 725 (48%) | 725 (58%) |  |
| Missing | 117 | 38 | 52 | 27 |  |
| **Self-rated physical health** |  |  |  |  | 0.5 |
| Excellent/Very Good | 1,607 (43%) | 373 (42%) | 665 (43%) | 569 (45%) |  |
| Good | 1,240 (33%) | 310 (35%) | 512 (33%) | 418 (33%) |  |
| Fair/Poor | 863 (23%) | 198 (22%) | 378 (24%) | 287 (23%) |  |
| Missing | 3 | 0 | 0 | 3 |  |
| **Depression screen (above cutoff)** |  |  |  |  | <0.001 |
| Below Cutoff (No) | 2,992 (83%) | 658 (77%) | 1,245 (83%) | 1,089 (88%) |  |
| Above Cutoff (Yes) | 593 (17%) | 193 (23%) | 252 (17%) | 148 (12%) |  |
| Missing | 128 | 30 | 58 | 40 |  |
| **Resilience** |  |  |  |  | 0.012 |
| High (Agree) | 2,262 (64%) | 498 (60%) | 931 (63%) | 833 (67%) |  |
| Neutral | 886 (25%) | 225 (27%) | 381 (26%) | 280 (23%) |  |
| Low (Disagree) | 401 (11%) | 108 (13%) | 168 (11%) | 125 (10%) |  |
| Missing | 164 | 50 | 75 | 39 |  |
| *^1^*Mean (SD); n (%) | | | | | |
| *^2^*One-way analysis of means (not assuming equal variances); Pearson's Chi-squared test | | | | | |

*^3^*Sex assigned at birth

**Supplementary Figure S2. Prevalence of unmet health and social service needs among White participants and participants of color (POC).** Bars represent the percentage of participants within white and participant of color groups who reported an unmet need for each service without reporting use of that service during the same period.

**Supplementary Table S3. Missingness covariate data and multiple-imputation methods in the primary analytic cohort (N = 3,713).** Missing covariate values were addressed using multiple imputation by chained equations (MICE). The primary outcome and chronological age were not imputed. Twenty imputed datasets were generated using 20 iterations.

| **Variable** | **Missing, N (%)** | **Imputation model** |
| --- | --- | --- |
| Any unmet service need | 0 (0.0%) | Not imputed |
| Chronological age (per 10 years) | 0 (0.0%) | Not imputed |
| Gender identity | 7 (0.2%) | Multinomial logistic regression |
| Race/ethnicity | 3 (0.1%) | Logistic regression |
| HIV status | 145 (3.9%) | Multinomial logistic regression |
| Financial insecurity | 18 (0.5%) | Logistic regression |
| Housing stability | 14 (0.4%) | Logistic regression |
| Transportation barriers | 99 (2.7%) | Logistic regression |
| Caregiving/support network | 156 (4.2%) | Logistic regression |
| Comfort disclosing SOGI | 255 (6.9%) | Multinomial logistic regression |
| Lifetime trauma | 117 (3.2%) | Logistic regression |
| Self-rated physical health | 3 (0.1%) | Multinomial logistic regression |
| Depression screen | 128 (3.4%) | Logistic regression |
| Resilience | 164 (4.4%) | Multinomial logistic regression |
|  | | |

**Supplementary Table S4. Association between chronological age and any unmet health or social service need across sequential Equitable Aging in Health Framework (EAHF) models.** Odds ratios represent the change in odds of any unmet need per 10-year increase in age. Covariates were added sequentially according to the EAHF. Estimates were pooled across 20 multiply imputed datasets.

| **Model** | **Age OR (95% CI)** | **P-value** | **Change in age coefficient from prior model, %** |
| --- | --- | --- | --- |
| **Model 1: Age only** | 0.90 (0.83–0.97) | 0.008 | -- |
| **Model 2: + Intersecting positionalities** | 0.96 (0.88–1.04) | 0.297 | 59.2 |
| **Model 3: + Financial insecurity** | 1.09 (0.99–1.19) | 0.069 | 285.6 |
| **Model 4: + Housing/transportation** | 1.07 (0.98–1.18) | 0.118 | -12.3 |
| **Model 5: + Caregiving/support** | 1.04 (0.95–1.14) | 0.388 | -43.9 |
| **Model 6: + Domains of power** | 1.07 (0.97–1.17) | 0.181 | 56.5 |
| **Model 7: + Health status** | 1.08 (0.98–1.19) | 0.100 | 26.0 |
| ORs represent the change in odds of any unmet service need per 10-year increase in age. Missing covariate values were addressed using multiple imputation by chained equations. The change in age coefficient is a descriptive percent change in the pooled log-odds coefficient relative to the immediately preceding model. AIC/BIC are not reported because they are not directly pooled by Rubin's rules. | | | |

**Supplementary Table S5. Adjusted predicted probabilities of any unmet health or social service need from the fully adjusted Equitable Aging in Health Framework (EAHF) model.** Predicted probabilities and 95% confidence intervals were estimated using average marginal predictions pooled across 20 multiply imputed datasets. Omnibus p-values were obtained by using pooled D1 tests comparing the final model with a model excluding the predictor.

| **Level** | **Adjusted probability, % (95% CI)** | **p-value** |
| --- | --- | --- |
| **Gender identity** | | |
| Cisgender man | 36.2 (34.3, 38.2) | 0.331 |
| Cisgender woman | 36.2 (33.6, 38.8) |  |
| TGD / gender expansive | 39.7 (35.3, 44.1) |  |
| **Race/ ethnicity** | | |
| POC | 40.6 (37.5, 43.7) | 0.004 |
| White | 35.6 (34.0, 37.1) |  |
| **HIV status** | | |
| HIV- | 35.2 (33.7, 36.8) | 0.001 |
| HIV+ | 43.0 (39.2, 46.8) |  |
| Unknown | 35.6 (23.9, 47.2) |  |
| **Financial insecurity** | | |
| No | 31.0 (29.3, 32.7) | <0.001 |
| Yes | 53.0 (49.5, 56.5) |  |
| **Housing stability** | | |
| No | 42.9 (38.4, 47.4) | 0.002 |
| Yes | 35.7 (34.2, 37.2) |  |
| **Transport barriers** | | |
| Yes | 52.4 (46.3, 58.6) | <0.001 |
| No | 35.3 (33.8, 36.8) |  |
| **Caregiving/ support network** | | |
| No | 36.0 (34.6, 37.5) | 0.011 |
| Yes | 43.0 (37.7, 48.3) |  |
| **SOGI disclosure** | | |
| Comfortable | 36.5 (35.0, 37.9) | 0.662 |
| Neutral | 36.5 (29.6, 43.3) |  |
| Uncomfortable | 39.4 (33.1, 45.8) |  |
| **Lifetime trauma** | | |
| No | 33.9 (31.9, 36.0) | <0.001 |
| Yes | 39.3 (37.2, 41.3) |  |
| **Depression screen (PHQ2)** | | |
| Above Cutoff | 46.5 (42.3, 50.7) | <0.001 |
| Below Cutoff | 34.6 (33.1, 36.2) |  |
| **Physical health** | | |
| Very Good | 31.2 (28.9, 33.5) | <0.001 |
| Fair/Poor | 46.1 (42.7, 49.6) |  |
| Good | 36.7 (34.2, 39.1) |  |
| **Resilience** | | |
| High | 34.9 (33.1, 36.8) | 0.019 |
| Low | 38.4 (33.9, 42.9) |  |
| Neutral | 39.9 (37.0, 42.8) |  |
|  | | |

**Supplementary Figure S3. Restricted cubic spline showing the association between chronological age and the predicted probability of any unmet health or social service need.** The solid line represents model-predicted probabilities, and the shaded region represents the 95% confidence interval. Restricted cubic spline models demonstrated no evidence of a nonlinear association between age and unmet need (p for nonlinearity=0.207).

**Supplementary Table S6. Multiple negative binomial regression of the number of unmet health and social service needs.** Incidence rate ratios (IRRs) and 95% confidence intervals were pooled across 20 multiply imputed datasets.

| **Characteristic** | **IRR (95% CI)** | **p-value** |
| --- | --- | --- |
| Age (per 10-year increase) | 1.07 (1.00–1.15) | 0.044 |
| Gender identity: Cisgender woman vs Cisgender man | 1.02 (0.88–1.15) | 0.767 |
| Gender identity: TGD / gender expansive vs Cisgender man | 1.12 (0.94–1.34) | 0.188 |
| Race/ethnicity: POC vs White | 1.13 (0.99–1.28) | 0.068 |
| HIV status: Positive vs Negative | 1.37 (1.19–1.59) | <0.001 |
| HIV status: Unknown vs Negative | 1.31 (0.90–1.91) | 0.154 |
| Financial insecurity (past 12 months): Yes/Maybe vs No | 2.14 (1.89–2.43) | <0.001 |
| Housing stability: Insecure/Unhoused vs Stably Housed | 1.50 (1.30–1.73) | <0.001 |
| Transportation barrier frequency: Frequent vs Rare/Infrequent | 1.75 (1.48–2.06) | <0.001 |
| Caregiving/support network: Yes vs No | 1.05 (0.88–1.27) | 0.570 |
| Comfort disclosing SOGI to provider: Neutral vs Comfortable | 1.28 (1.00–1.64) | 0.053 |
| Comfort disclosing SOGI to provider: Uncomfortable vs Comfortable | 1.35 (1.08–1.68) | 0.008 |
| Lifetime trauma experience: Yes vs No | 1.22 (1.09–1.37) | <0.001 |
| Self-rated physical health: Good vs Excellent/Very Good | 1.39 (1.21–1.59) | <0.001 |
| Self-rated physical health: Fair/Poor vs Excellent/Very Good | 1.99 (1.71–2.31) | <0.001 |
| Depression screen (above cutoff): Above cutoff vs Below cutoff | 1.46 (1.27–1.68) | <0.001 |
| Resilience: Neutral vs High | 1.34 (1.17–1.53) | <0.001 |
| Resilience: Low vs High | 1.28 (1.08–1.52) | 0.005 |
|  | | |
